# Complete Genome Sequence of *Spiroplasma litorale* TN-1^T^ (DSM 21781), a Bacterium Isolated from a Green-Eyed Horsefly (*Tabanus nigrovittatus*)

**DOI:** 10.1128/genomeA.01116-15

**Published:** 2015-10-01

**Authors:** Wen-Sui Lo, Yi-Ching Lai, Yun-Wei Lien, Tzu-Haw Wang, Chih-Horng Kuo

**Affiliations:** a Institute of Plant and Microbial Biology, Academia Sinica, Taipei, Taiwan; b Molecular and Biological Agricultural Sciences Program, Taiwan International Graduate Program, National Chung Hsing University and Academia Sinica, Taipei, Taiwan; c Graduate Institute of Biotechnology, National Chung Hsing University, Taichung, Taiwan; d Biotechnology Center, National Chung Hsing University, Taichung, Taiwan

## Abstract

*Spiroplasma litorale* TN-1^T^ (DSM 21781) was isolated from the gut of a green-eyed horsefly (*Tabanus nigrovittatus*), collected at Ocracoke Island in North Carolina in 1983. Here, we report the complete genome sequence of this bacterium to facilitate the investigation of its biology.

## GENOME ANNOUNCEMENT

*Spiroplasma litorale* is a bacterium known to be associated with tabanid flies belonging to the genus *Tabanus* (1). Strains assigned to this species have been isolated from *Tabanus* species *T. americanus*, *T. atratus*, *T. gladiator*, *T. lineola*, *T. nigrovittatus*, *T. sulcifrons*, and *T. zythicolor*. The restriction of *S. litorale* to the coastal areas of the southeastern United States makes it one of the best-known examples of a geographically limited insect symbiont within the genus *Spiroplasma* (1). To facilitate the future investigation on the biology of this bacterium and to improve the taxon sampling of available *Spiroplasma* sequences for comparative genomics and evolutionary studies, we determined the complete genome sequence of *S. litorale* TN-1^T^.

The strain was acquired from the German Collection of Microorganisms and Cell Cultures (catalogue no. DSM 21781). The freeze-dried sample was processed according to the manufacturer’s instructions and cultured in the M1D medium (2) prior to DNA extraction using the Wizard genomic DNA purification kit (Promega, USA). PCR and Sanger sequencing were performed to verify that the 16S rRNA gene sequence matched the reference record (GenBank accession no. NR_025708.1).

The procedures for sequencing, assembly, and annotation were based on those described in our previous studies on *Spiroplasma* genomes (3–8). Briefly, the Illumina MiSeq platform was used to generate 300-bp reads from one paired-end library (~570-bp insert, 2,155,514 reads) and one mate-pair library (~4,000-bp insert, 2,024,260 reads). The initial *de novo* assembly was performed using ALLPATHS-LG release 52188 (9). Subsequently, PAGIT version 1 (10) was used to assist an iterative process for improving the assembly. For each iteration, the raw reads were mapped to the assembly using Burrows-Wheeler Aligner (BWA) version 0.7.12 (11), programmatically checked using the MPILEUP program in the SAMTools package version 1.2 (12), and visually inspected using IGV version 2.3.57 (13). Polymorphic sites and gaps were corrected based on the mapped reads. The process was repeated until the complete genome sequence was obtained.

The programs RNAmmer (14), tRNAscan-SE (15), and Prodigal (16) were used for gene prediction. The gene names and product descriptions were first annotated based on the homologous genes in other *Spiroplasma* genomes (3–8), as identified by OrthoMCL (17). Subsequent manual curation was based on BLASTp (18) searches against the NCBI nonredundant database (19) and the KEGG database (20, 21).

The circular chromosome of *S. litorale* TN-1^T^ is 1,225,519 bp in size and has a G+C content of 24.9%; no plasmid was found. The first version of annotation includes one set of 16S-23S-5S rRNA genes, 29 tRNA genes (covering all 20 amino acids), and 1,064 protein-coding genes.

### Nucleotide sequence accession number. 

The complete genome sequence of *S. litorale* TN-1^T^ has been deposited at DDBJ/EMBL/GenBank under the accession no. CP012357.
